# The Value of Vaccination

**Published:** 1884-11

**Authors:** 


### The Value of Vaccination.

That there are still intelligent people who oppose vaccination, and strive to make it appear that it is not only useless but injurious, need surprise no one aquainted with the vagaries of the human mind. For such persons, testimony is of no avail. They are not capable of seeing the conclusions of a logical train of reasoning. Facts to them are inferior in power to prejudices. Yet there are facts which now and then are brought to one's notice, so startling in their native hideousness that it seems wrong to pass them over in silence. If it is only a matter of medical statistics, we must print a reference to a letter from Dr. Neve of the Mission Hospital in Cashmere, which has appeared in the *Civil and Military Gazette of Lahore.* "Thanks to the exertions of the English authorities, vaccination has been carried to some extent in that portion of India ruled by us; but in Cashmere the state of things in an entirely unprotected country was to be seen." Dr. Neve says it would be nearer the truth to say that the population is annihilated than to say that it is decimated by the scourge of small-pox. Small-pox is endemic in every village and town of Cashmere. "I recently obtained from all my hospital staff a statement of the mortality of small-pox among their immediate relatives. They represent twenty-five families, and in these 190 members were born, of whom exactly 100 died of small-pox. Two or three children have not yet been attacked; all others have had the disease." Thus, of these 190 persons, at least 95 per cent. had been attacked by small-pox, and of those 65 per cent. succumbed. "There is not much room for hoping," Dr. Neve says, "that these figures indicate any very unusual rate of mortality, and, of course, the evils inflicted by the disease are lifelong in many who survive the attack." Such is the condition of things in a country where vaccination is not practiced, and such it was here before the discovery of Jenner. So it would be again were the crazy notions of the anti-vaccinationists to prevail—which, however, we do not greatly fear. The world may be old, but it is not that senile.

An iodoform collodion is well spoken of, consisting of iodoform ten parts, collodion ninety parts. To be applied with a camel's-hair brush, the edges of the wound being held together until it dries. For wounds in exposed situations.

